# AI-driven radiogenomics in gynecologic oncology: from radiological digital biopsy to a new paradigm in precision therapy

**DOI:** 10.3389/fonc.2026.1745519

**Published:** 2026-02-04

**Authors:** Qiqi Kong, Yunqing Ban

**Affiliations:** Department of Radiology, The Fifth Affiliated Hospital of Xinjiang Medical University, Urumqi, China

**Keywords:** artificial intelligence, digital biopsy, foundation models, gynecologic oncology, radiogenomics, tumor heterogeneity

## Abstract

Tumor heterogeneity is a core challenge in gynecologic oncology, driving therapeutic resistance and limiting the efficacy of single-point biopsies. Artificial intelligence (AI) and radiomics are emerging as a “digital biopsy” to non-invasively decode tumor biology from medical radiological modalities images(including MRI, CT, and PET). This review synthesizes the state of AI in predicting key molecular features across gynecologic cancers, including homologous recombination deficiency (HRD) in ovarian cancer, microsatellite instability (MSI) and PI3K activation in endometrial cancer, and, as an illustrative case, HPV integration and DNA methylation in cervical cancer. We further explore how advanced architectures like Vision Transformers (ViTs) and Graph Neural Networks (GNNs) can delineate the tumor microenvironment and predict therapeutic response. Finally, we discuss critical hurdles to clinical translation—such as model generalizability, the need for causal AI, and the data bottleneck—while examining future paradigms like foundation models and patient-specific “digital twins.” This review highlights AI’s revolutionary potential to link imaging phenotype with molecular genotype, advancing a new era of precision medicine in gynecologic oncology.

## 1 Introduction

One of the central challenges in clinical oncology stems from the fundamental nature of cancer: evolution (1, 2). A tumor is not a homogenous mass of cells but a complex, dynamically evolving ecosystem populated by subclones with diverse genotypes and phenotypes (3, 4). This profound intratumor heterogeneity (ITH) is a significant contributing factor to therapeutic resistance, disease relapse, and metastasis (5–7). Consider a clinical scenario: a 58-year-old woman with endometrial cancer whose single-site biopsy reveals a G2 endometrioid adenocarcinoma, p53 wild-type (8). This sample, however, may represent only a fraction of the tumor landscape, missing a distant, more aggressive p53-mutant subclone, thereby leading to suboptimal treatment decisions and a poor outcome (8–10). This scenario precisely exposes the fundamental limitation of the current “gold standard” reliance on single-point, invasive biopsies: inherent spatial sampling bias (8, 10). A particularly striking example is cervical cancer, where the HPV genome integrates into the host chromosome.—a key event in malignant progression—can be highly heterogeneous across the tumor, making it difficult to assess comprehensively with a small tissue sample (10).

Gynecologic cancers, primarily ovarian, endometrial, and cervical cancers, are a major threat to women’s health globally (11, 12). Thanks to large-scale sequencing initiatives like The Cancer Genome Atlas (TCGA), our understanding of their molecular landscapes has expanded exponentially (10, 13, 14). These studies have directly linked specific molecular features to clinical decisions. For instance, in high-grade serous ovarian cancer (HGSOC), Homologous Recombination Deficiency (HRD) status is a critical biomarker for Poly(ADP-ribose) polymerase inhibitors (PARPi) therapy (12, 15, 16). In endometrial cancer, a molecular classification system profoundly changes prognostic stratification and treatment strategies (17–19). Notably, mismatch repair deficiency (dMMR), or its surrogate high microsatellite instability (MSI-H), is a “pan-cancer” biomarker for immune checkpoint inhibitors (ICIs) (20–22). However, obtaining this molecular information remains a challenge. Tissue biopsies see the “trees” but miss the “forest,” while liquid biopsies lack spatial information (23, 24). This dilemma has created a critical unmet need for tools that can non-invasively characterize the entire tumor.

It is against this backdrop that the convergence of radiomics and artificial intelligence (AI) has emerged (25, 26). The core scientific hypothesis is that a tumor’s molecular genotype drives microscopic biological changes that manifest on macroscopic medical images as unique, quantifiable imaging phenotypes (25, 27, 28). This “digital biopsy” approach aims to decode the tumor’s biology directly from voxels, bridging the gap between genotype and phenotype. This concept forms the basis of the field of radiomics, which aims to bridge medical imaging and personalized medicine (26). We will explore how AI decodes key molecular markers, with a special focus on using cervical cancer to illustrate how AI can predict specific molecular events like HPV integration and DNA methylation, thereby showcasing the full potential of the digital biopsy paradigm (29, 30). The subsequent sections will delve into specific molecular markers, including the aforementioned HPV-driven events and DNA methylation patterns, to build a robust case for this transformative (31).

### 1.1 Search strategy and selection criteria

**Figure 1:**
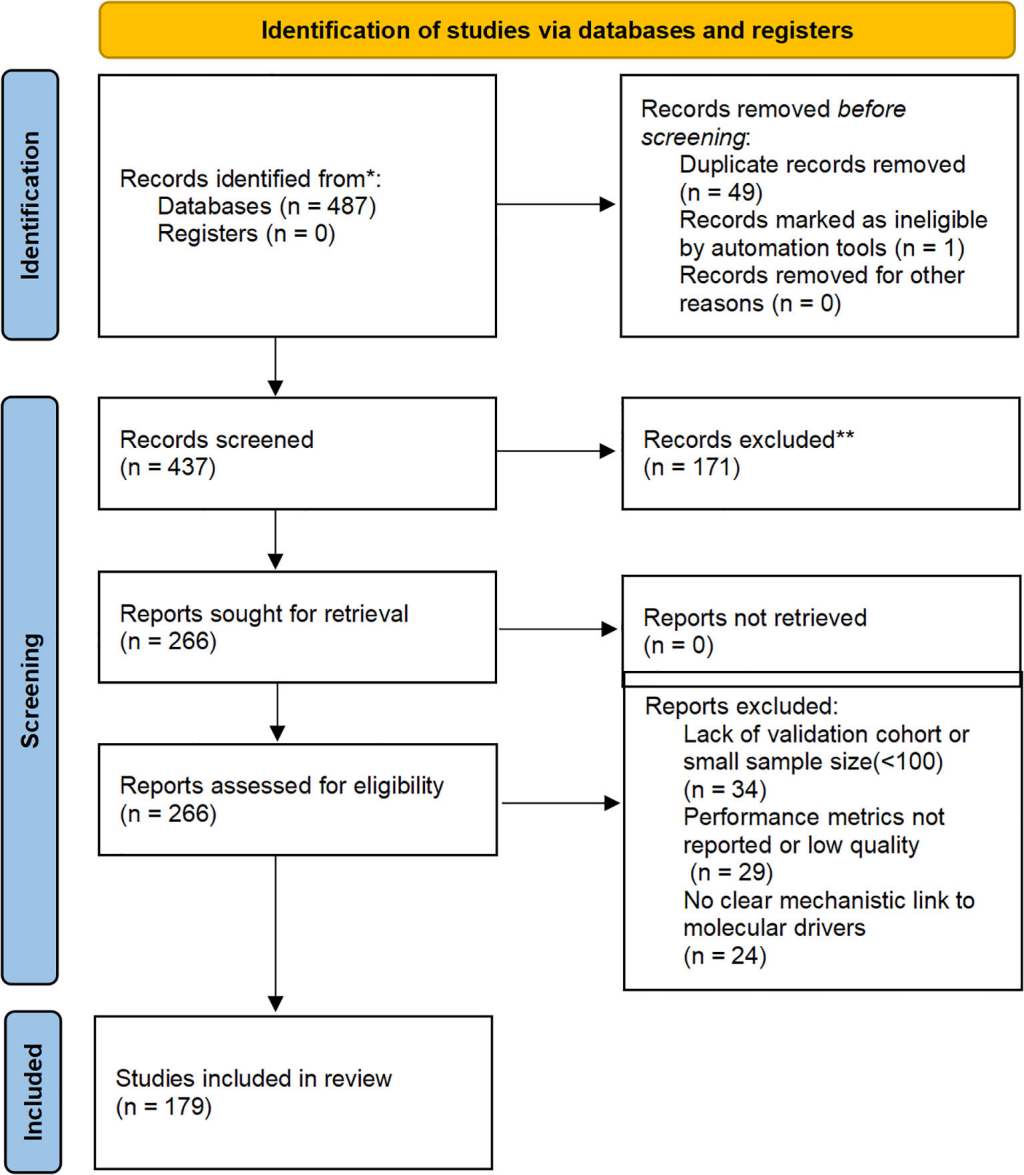
Flow diagram of the study selection process based on the PRISMA 2020 guidelines..

To enhance transparency in our narrative review, we conducted a structured literature search across PubMed, Scopus, Web of Science, and Google Scholar from January 2020 to October 2025. Search terms included combinations such as “AI gynecologic oncology,” “radiogenomics ovarian cancer,” “digital biopsy cervical cancer,” and “molecular imaging endometrial cancer.” Inclusion criteria focused on peer-reviewed articles applying AI/deep learning to radiological imaging (CT, MRI, PET) or digital pathology for molecular profiling in ovarian, endometrial, or cervical cancers, with at least retrospective validation (≥100 patients preferred), performance metrics (e.g., AUC ≥0.70), and explicit mechanistic links to drivers like HRD or MSI. From 487 initial records, duplicates were removed (n=50 excluded), titles/abstracts screened (n=171 excluded), and full-texts assessed (n=87), yielding 179 key studies for synthesis. The process is illustrated in **Figure 1**.

## 2 The AI toolkit: from feature engineering to application-driven architectures

Transforming medical images into quantitative biological probes relies on a powerful AI toolkit, moving from hypothesis-driven feature engineering to application-driven deep learning architectures (32–34). Utilizing the advanced AI architectures discussed below, researchers are now able to probe the molecular underpinnings of gynecologic cancers with unprecedented, non-invasive depth.

### 2.1 The classical radiomics workflow

**Figure 2:**
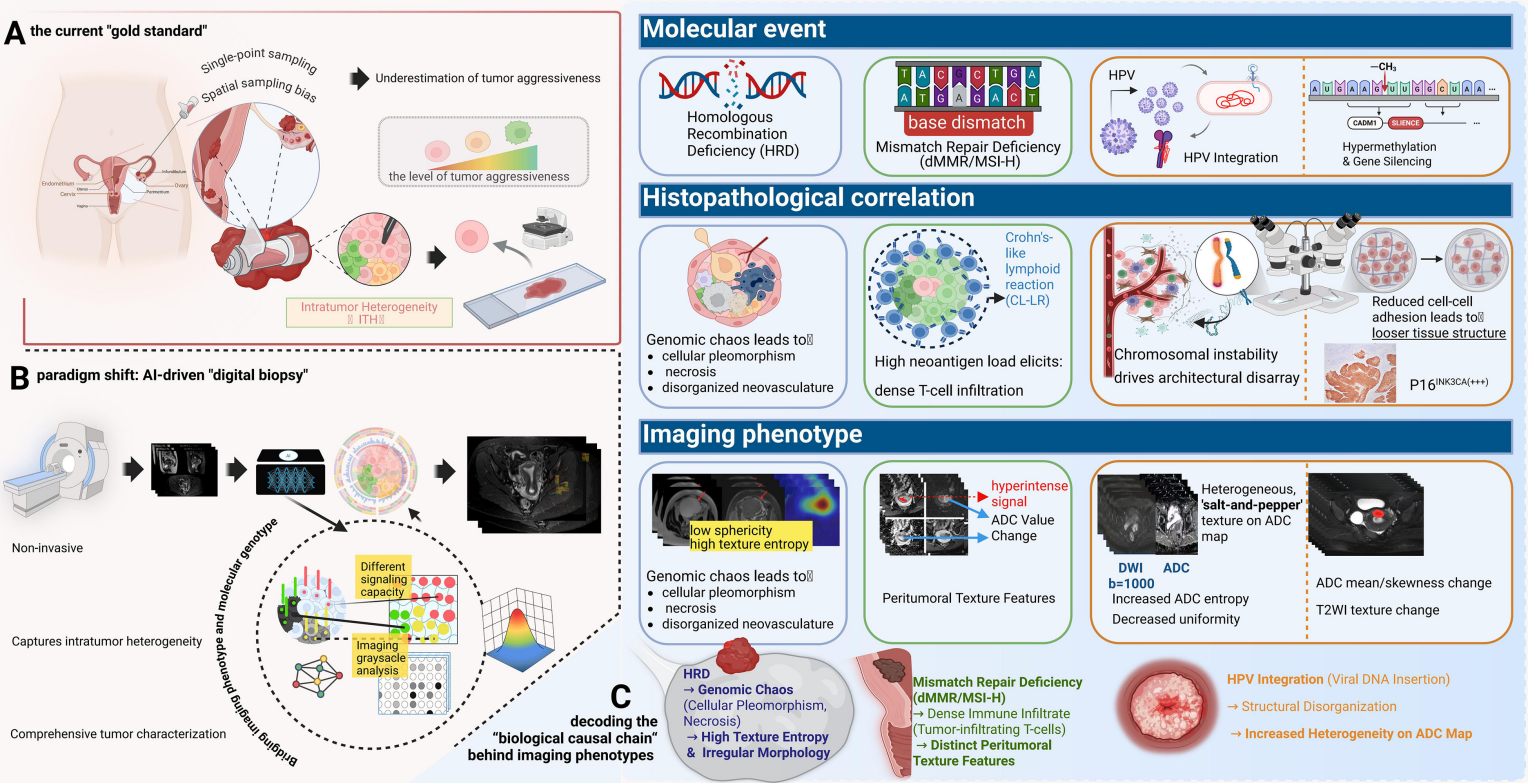
The paradigm shift to “digital biopsy” and the biological causal chain underlying imaging phenotypes. **(A)** Limitations of Physical Biopsy: The current “gold standard” relies on single-point sampling, which is prone to spatial bias and often underestimates tumor aggressiveness. **(B)** AI-Driven Digital Biopsy: A non-invasive approach that captures comprehensive intratumor heterogeneity across the entire tumor volume. **(C)** Decoding the Biological Causal Chain: This panel links molecular events to imaging signatures: HRD induces genomic chaos and necrosis, manifesting as high texture entropy and irregular morphology; dMMR/MSI-H triggers immune infiltration (e.g., Crohn’s-like lymphoid reaction), resulting in distinct peritumoral texture features; and HPV integration drives structural disorganization, reflected as a heterogeneous “salt-and-pepper” texture on ADC maps.

The traditional radiomics pipeline is a multi-step process, involving standardized image acquisition, reproducible segmentation, high-throughput extraction of IBSI-compliant features (shape, texture, wavelet) (35). In gynecologic oncology, these features serve as quantitative proxies for heterogeneity; for instance, high-order texture features (e.g., GLCM entropy) may reflect the chaotic micro-architecture of high-grade serous ovarian cancer (27, 64). Finally, robust feature selection (e.g., LASSO) to train machine learning models (36) (**Figure 2**).

### 2.2 Deep learning: the end-to-end autonomous learning paradigm

Deep learning (DL) represents a fundamental shift (37), using deep neural networks (DNNs) to learn relevant features automatically from raw pixels (38, 39).

#### 2.2.1 Convolutional neural networks

As the cornerstone of image analysis (40), architectures like U-Net are now standard for cervical tumor segmentation on MRI (41), while ResNet and DenseNet (42, 43) variants effectively distinguish benign from malignant adnexal masses by learning hierarchical feature representations (174).

#### 2.2.2 Vision transformers

ViTs treat an image as a sequence of patches and use a self-attention mechanism to model long-range, global dependencies (44, 45). This global context modeling is particularly powerful for analyzing the tumor invasive front, a complex micro-anatomical structure whose features are strong predictors of cancer progression (46, 47). By assessing the entire tumor boundary simultaneously, ViTs are uniquely positioned to identify subtle patterns of invasion in cervical cancer that are missed by the limited receptive fields of CNNs (47, 48).

#### 2.2.3 Graph neural networks

GNNs model data as a graph of nodes and edges (49) making them ideal for capturing relationships (50). This is uniquely suited for modeling the tumor microenvironment (TME) (51). For instance, in endometrial cancer, individual cells (cancer, immune, stromal) can be modeled as ‘nodes’ and their spatial adjacencies as ‘edges.’ This topology-aware approach allows the GNN to quantify the interplay between tumor-infiltrating lymphocytes and cancer cells—such as the Crohn’s-like lymphocytic response—which is a key predictor of MSI status and immunotherapy response (52).

### 2.3 The paradigm shift towards foundation models

The data bottleneck remains a primary challenge (38). A paradigm shift is underway from task-specific models to Foundation Models (53). Pre-trained on vast, unlabeled datasets using self-supervised learning, these models learn rich, generalizable representations of medical images (54–56). They can then be fine-tuned for specific tasks (e.g., HRD prediction) with very few labeled examples (“few-shot learning”), representing a promising solution to the data scarcity problem in medical imaging. complexity of a tumor cannot be fully captured by a single data source (56, 57). Multi-modal AI aims to build more comprehensive models by fusing data from imaging, digital pathology, and genomics (e.g., *PIK3CA*, *KRAS* mutations) (58, 59). This enables a more holistic view of the tumor’s biological state, leading to more precise predictions (59, 60).

## 3 Decoding key molecular signatures: from broad correlations to a mechanistic digital biopsy

The transformative potential of AI lies in its ability to non-invasively predict clinically critical molecular features (**Table 1**).

**Table 1:** Application cases, mechanistic links, predictive accuracies, and clinical values for AI molecular imaging in gynecologic cancers.

Core molecular event/biological process	Key genomic/epigenetic drivers	Affected cancer& freq.	Key histopathological correlate	AI-driven imaging phenotype hypothesis	Clinical readiness	Predictive accuracy	Clinical value
Homologous Recombination Deficiency, (HRD)	*BRCA1/2* mutations, etc. (15, 61)	Ovarian (HGSOC, ~50%) (12)	High clonal diversity, pleomorphism, necrosis, disorganized neovasculature (64).	Genomic chaos manifests as increased textural heterogeneity and irregular morphology on CT/MRI (66)	Level 2: Retrospective Validation	AUC 0.81 (DeepHRD model on H&E slides in TCGA cohort) (175)	Correlates with better OS after platinum therapy (HR 0.46); identifies 3.1-fold more HRD cases for PARP inhibitors, improving targeted treatment response (175).
Mismatch Repair Deficiency (dMMR)/MSI-H	*MLH1, MSH2* silencing, etc. (22)	Endometrial (~25-30%) (14)	Dense lymphocyte infiltration, often with a “Crohn’s-like” peritumoral response (88)	High lymphocyte infiltration alters tissue density, creating distinct peritumoral and internal textures on MRI (74, 75).	Level 2: Retrospective Validation	Accuracy 94% (G1G2 endometrioid) and 84% (G3); sensitivity up to 100% (deep learning on H&E slides) (176)	Predicts immunotherapy response (e.g., pembrolizumab; rates 44-57% in *MSI-H* EC), enhancing PFS/OS in advanced cases (176).
PI3K/AKT/mTOR Pathway Dysregulation	*PIK3CA, PTEN* alterations (89)	Endometrial (~80%), Cervical (~50%) (10, 14)	Increased cellular proliferation, metabolic reprogramming (Warburg effect), and angiogenesis	Increased glucose metabolism (high PET SUV) and vascularity (high DCE-MRI perfusion) are detectable.	Level 2: Retrospective Validation	Sensitivity 73-84%, specificity 91-95% (ICC for Akt/mTOR in cytology; limited direct AI imaging data) (177)	Links to targeted therapies (e.g., mTOR inhibitors); upregulation correlates with aggressive EC progression and poorer OS (177).
HPV Integration & E6/E7 High Expression	Disruption of HPV *E2* gene (79, 90)	Cervical (>99%) (10)	Chromosomal instability and architectural disarray driven by oncoprotein expression (10).	Viral-driven genomic instability creates more chaotic textural (entropy) and diffusion (ADC) patterns on MRI.	Level 1: Preclinical Hypothesis	Higher sensitivity/specificity than Pap cytology (automated dual-stain AI reduces colposcopy referrals by ~33%) (178)	Improves early detection in HPV-positive cases, leading to better PFS/OS through timely intervention (178).
Specific DNA Methylation (e.g., *CADM1*)	Epigenetic silencing of tumor suppressors (83, 84)	Cervical (High-grade lesions) (80)	Reduced cell-cell adhesion and altered apoptosis rates, leading to looser tissue structure.	Micro-architectural changes alter water mobility, detectable as shifts in ADC values and T2WI texture.	Level 1: Preclinical Hypothesis	Sensitivity 83.3%, specificity 95.5% (combined *CADM1*/*MAL* in liquid biopsy; no direct AI imaging reported) (179)	Serves as biomarker for early high-grade lesion detection; correlates with progression risk and potential PFS benefits via monitoring (179).

Clinical Readiness Levels: Level 1: Preclinical Hypothesis (Theoretical basis with preliminary in vitro/in vivo evidence); Level 2: Retrospective Validation (Validated in retrospective cohorts); Level 3: Prospective Validation (Validated in prospective studies or clinical trial subgroups); Level 4: Clinical Integration (Incorporated into clinical guidelines or routine decision support) (91).

PFS, progression-free survival; OS, overall survival.

### 3.1 Ovarian cancer: deconstructing the “genomic chaos” of HRD biological rationale

The core causal chain is as follows: The molecular event of Homologous Recombination Deficiency (HRD) (61) leads to the genomic consequence of an inability to precisely repair DNA double-strand breaks. This results in the accumulation of large-scale structural variants known as “genomic scars” (loss of heterozygosity [LOH], telomeric allelic imbalance [TAI], and large-scale state transitions [LST]) (62–64). This profound genomic instability serves as an engine for rapid clonal evolution, leading to the key histopathological correlate of significant cellular pleomorphism, areas of necrosis (as some clones outgrow their blood supply), and disorganized, leaky neovasculature (64). This structural chaos directly translates into a measurable imaging phenotype. On CT/MRI, this is observed as irregular tumor morphology, central non-enhancing regions (necrosis), and heterogeneous, avid contrast enhancement (disorganized vasculature) (65). Therefore, radiomic features quantifying texture heterogeneity (e.g., entropy) and shape irregularity (e.g., low sphericity) are direct surrogates for the underlying biological state of HRD. Current Research & Critical Assessment: Numerous studies have built models to predict HRD or BRCA status based on these principles (66, 67). However, a key challenge is distinguishing the imaging phenotype of BRCA-mutated HRD from non-BRCA HRD, which may have different biological underpinnings (68). Furthermore, predicting ubiquitous mutations like TP53 remains a complex task, though it is often linked to features of necrosis and architectural disarray (69).

### 3.2 Endometrial cancer: linking MSI to histopathological immune signatures biological rationale

The molecular event of Mismatch Repair Deficiency (dMMR) leads to high microsatellite instability (MSI-H) (17). The immunological consequence is the accumulation of frameshift mutations, creating a high neoantigen load and rendering the tumor highly immunogenic (70). This elicits the key histopathological correlate of dense infiltration by cytotoxic T-lymphocytes and other immune cells, often organized into a distinct “Crohn’s-like lymphocytic response” at the tumor’s invasive margin (71). This physical immune barrier alters the tumor-stroma interface and tissue density. This leads to the imaging phenotype hypothesis that this dense peritumoral immune reaction is visible on MRI, manifesting as unique peritumoral signals (e.g., enhancement or T2 signal changes) (72). The altered internal cellular composition (mix of tumor and immune cells) also changes diffusion properties (ADC values) and texture compared to the typically “immune-desert” microsatellite stable (MSS) tumors (73).AI models have shown high accuracy in predicting MSI status from MRI (74, 75), and multi-modal models incorporating PET have further improved performance (76). These findings are analogous to successes in predicting MSI from digital pathology slides (77).

### 3.3 Cervical cancer: a flagship case for a mechanistic digital biopsy

As a disease with a clear viral etiology and a well-defined molecular progression, cervical cancer serves as the perfect model system to demonstrate the profound potential of the digital biopsy paradigm (10, 78).

#### 3.3.1 AI for predicting HPV integration (a “digital karyotype”) mechanistic link

The concept of “digital karyotyping” suggests that genomic instability manifests as distinct morphological phenotypes. In cervical cancer, HPV integration induces ‘genomic scars’ and chromosomal instability (10), which may lead to cellular pleomorphism and architectural disarray. We hypothesize that these microscopic changes alter water diffusion, allowing DWI-derived texture features to potentially serve as a macroscopic ‘digital karyotype’ of tumor aggressiveness (9, 81).The molecular event of HPV integration into the host genome frequently occurs at common fragile sites, leading to the genomic consequence of significant chromosomal instability and amplification of adjacent oncogenes like *MYC* (10, 79). This “genomic chaos” is a direct biological driver of ITH (9). The histopathological correlate is disordered tissue architecture and marked cellular pleomorphism (4). This structural disarray disrupts the uniform environment for water molecule diffusion, creating complex interfaces between cell populations, which translates to a measurable imaging phenotype of increased high-order texture features (higher entropy, lower uniformity in GLCM) and a more heterogeneous ADC map on MRI (80, 81).

The core hypothesis is that an AI model can learn these textural and diffusion signatures to non-invasively predict HPV integration status, providing a powerful risk stratification tool beyond simple HPV DNA detection (82).

#### 3.3.2 AI for predicting DNA methylation (a “digital methylome”) mechanistic link

The molecular event of hypermethylation and believes that the epigenetic state of the host tumor suppressor genes is one of the most important drivers of cervical cancer (83, 84). In recent years, the role of DNA methylation as an important indicator for the early detection of cervical cancer has been confirmed. For instance, methylation panels targeting key genes such as *ZSCAN1* and *ST6GALNAC5* (e.g., the WID-qCIN test), have been validated in large-scale, real-world studies as a highly effective triage tool, highlighting their growing importance (85). The cellular consequence of silencing these genes includes reduced cell-cell adhesion and evasion of apoptosis, allowing damaged cells to survive. Concurrently, a core protein-level biomarker is the overexpression of p16^INK4a^. The underlying mechanism is directly linked to the E7 oncoprotein of high-risk HPV types: the E7 protein interacts with and degrades the retinoblastoma protein (pRb), which lifts the negative feedback inhibition on the cyclin-dependent kinase inhibitor p16^INK4a^, leading to its massive intracellular accumulation. This serves as a hallmark event of HPV-driven cellular transformation (86). The histopathological correlate is a less cohesive, more disorganized tissue structure with an altered ratio of viable to apoptotic cells, changing the density of the xtracellular matrix. This directly impacts the imaging phenotype: these microstructural changes alter the mobility of water molecules. A looser structure may increase the extracellular water space, leading to higher ADC values, while uncontrolled proliferation could do the opposite. The heterogeneity of these processes across the tumor is captured by texture features on ADC maps and T2-weighted images.

The feasibility of predicting DNA methylation from MRI is no longer purely hypothetical. High-impact studies in other cancers (e.g., glioblastoma) have already demonstrated a strong correlation between global DNA methylation levels and specific radiomic features (87). The hypothesis is that a similar “digital methylome” model can be built for cervical cancer, elevating the digital biopsy from the genetic to the epigenetic level.

However, decoding these tumor cell-intrinsic molecular features is only the beginning of the story. A core concept in modern oncology is that a tumor is not merely a collection of malignant cells, but a complex and dynamically evolving community of cancer cells, immune cells, stromal cells, and vasculature (4). Therefore, to truly understand and ultimately control the tumor, we must achieve a “leap in scale”: from decoding the biology of the “single cancer cell” to delineating the complex biological behavior of the “entire tumor ecosystem.

## 4 Delineating the tumor microenvironment and guiding therapy

Building upon the decoding of cell-intrinsic molecular features from imaging in the previous section, this section elevates the perspective to a more macroscopic biological scale: the tumor microenvironment (TME). Beyond cell-intrinsic features, AI can characterize the broader TME.

### 4.1 Characterizing “cold” and “hot” immune landscapes

Tumors are broadly categorized into “hot” (inflamed) and “cold” (immune-desert) phenotypes, a critical distinction for immunotherapy (92, 93). For example, MSI-high endometrial cancers typically present as “hot” tumors with dense CD8+ T-cell infiltration, whereas many ovarian cancers exhibit a “cold”, immunosuppressive stroma.The rationale is that this immune infiltration alters tissue density and vascularity, creating a detectable radiomic signature—such as specific texture patterns at the tumor-stroma interface. AI models have successfully predicted ICI response in other cancers from CT scans (94). Similar work in gynecologic cancers is emerging (95).

### 4.2 Assessing tumor hypoxia and guiding “dose-painting” radiotherapy

Hypoxia drives treatment resistance (96, 97). Functional imaging can visualize hypoxic regions (98), and AI can generate voxel-level hypoxia maps. This is crucial for “dose-painting” radiotherapy, where radiation doses are adaptively escalated to the most resistant subregions (99, 100).

### 4.3 Delta-radiomics: gaining insight into early treatment response

RECIST 1.1 criteria are often a late indicator of response (101). Delta-radiomics analyzes the change (Δ) in radiomic features between baseline and early on-treatment scans (102, 103). Effective therapy induces rapid cellular changes that alter imaging textures long before tumor shrinkage (104, 105). This has shown great promise for early response assessment in ovarian and cervical cancer (105, 106).

### 4.4 AI in predicting radiotherapy toxicity: protecting the patient

While “dose-painting” focuses on escalating therapeutic effects, an equally critical clinical challenge is the mitigation of therapeutic harm. In gynecologic oncology, particularly for cervical and endometrial cancers, radiotherapy is a cornerstone of treatment, but it carries a significant risk of acute and late toxicity to surrounding organs at risk (OARs), such as the bladder and rectum, leading to conditions like radiation cystitis and proctitis that severely impact patient quality of life (107). Predicting which patients are at high risk for severe toxicity is a key unmet need for treatment individualization.

AI and machine learning models offer a powerful new solution (108, 109). By integrating multi-dimensional data, these models can build complex, non-linear predictive tools to identify high-risk patients before treatment initiation (110). The input features for these models are diverse, typically including: 1) *Clinical features* like patient age, BMI, comorbidities (e.g., Charlson Comorbidity Index), and performance status (KPS); 2) *Dosimetric features* extracted from dose-volume histograms (DVHs), such as the minimum dose to 2cc (D2cc of the rectum or bladder; and 3) *Radiomic features* from pre-treatment CT or MRI scans that quantify tissue texture and morphology of the OARs (111).

Current research has demonstrated the promise of this approach, with models based on Support Vector Machines (SVM), Random Forests, and other architectures achieving encouraging performance (often with an AUC > 0.7, considered clinically useful) in predicting grade 3 or higher toxicities (108, 112). However, echoing the challenges discussed in the next section, these models often suffer from a lack of generalizability and external validation, underscoring the critical need for standardized data collection and multi-institutional collaborative studies to translate this potential into a reliable clinical tool (108, 110, 113).

## 5 The path to clinical translation: from algorithms to clinical practice

**Figure 3:**
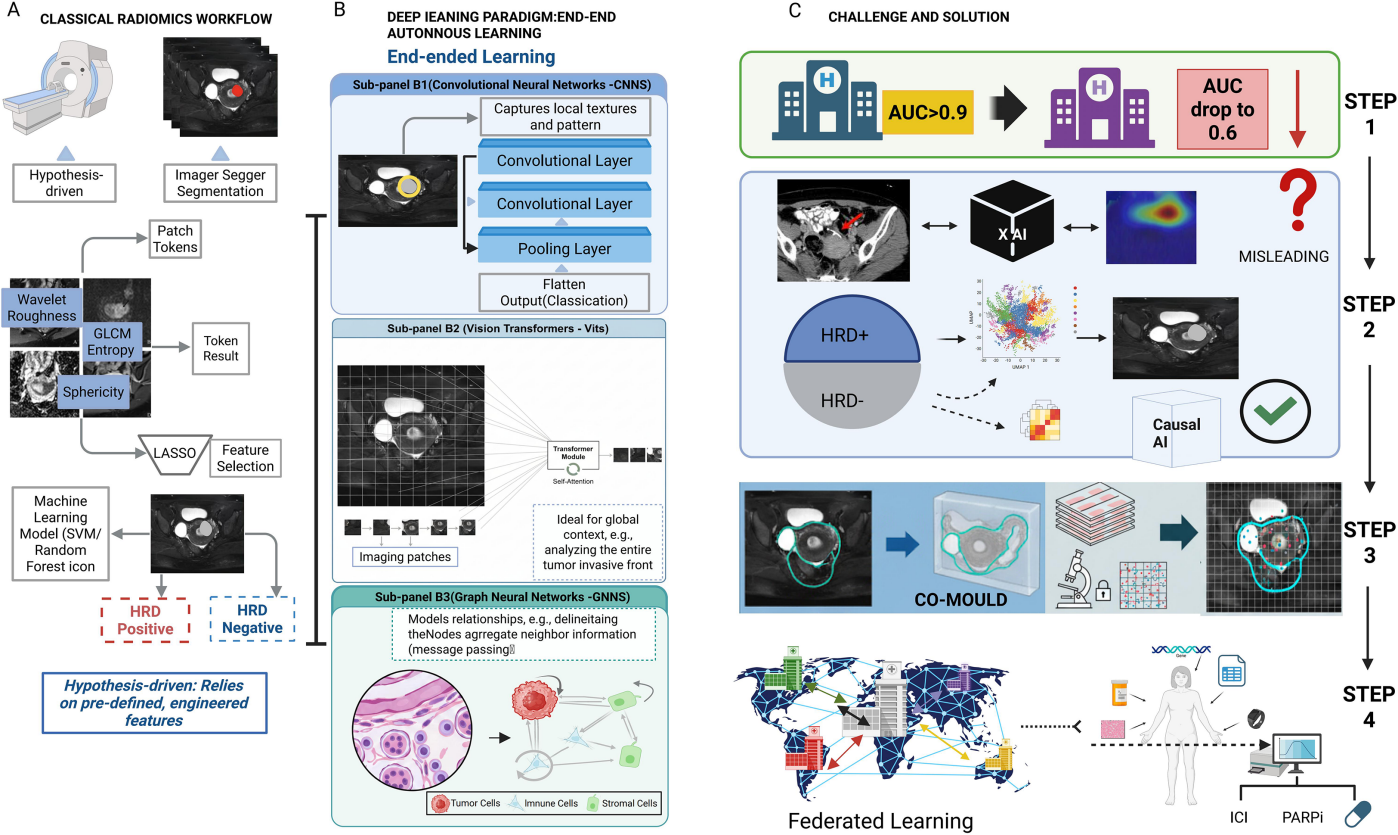
Comparison of classical radiomics versus deep learning paradigms and strategies for clinical translation. **(A)** Classical Radiomics Workflow: A hypothesis-driven pipeline involving tumor segmentation, extraction of handcrafted features (e.g., GLCM, wavelet), and feature selection (LASSO) to train machine learning classifiers. **(B)** Deep Learning Paradigm: An end-to-end autonomous learning approach comprising: (B1) CNNs for capturing local textures; (B2) Vision Transformers (ViTs) for encoding global context via self-attention; and (B3) Graph Neural Networks (GNNs) for modeling cellular topology and neighbor relationships. **(C)** Challenges and Solutions: The diagram outlines steps to bridge clinical gaps. Federated Learning addresses generalization issues (AUC drops). The CO-MOULD framework serves as a central engine for precise HRD stratification. Finally, Causal AI is integrated to filter misleading confounders, guiding targeted therapies like ICI and PARP inhibitors.

Despite immense promise, translating AI models into clinical practice faces significant hurdles (114) (**Figure 3**).

### 5.1 Generalizability and reproducibility: “from my data to your data”

Many models fail to generalize due to “dataset shift” (115, 116). While “underspecification” is a correct term (117), a more mechanistic explanation for these failures is the phenomenon of “Shortcut Learning” (118). Shortcut learning describes a model’s tendency not to learn the intended, biologically relevant causal features of a disease, but to instead seize upon spurious, non-generalizable correlations that happen to be associated with the label in the training data. For example, a model might learn to associate the presence of a surgical clip, text annotations on an image, or the unique noise profile of a specific scanner with a particular diagnosis, rather than the actual tumor morphology (119). When deployed on new data from a different institution lacking these “shortcuts,” the model’s performance collapses, revealing it never learned the underlying biology at all. This is a primary threat to model credibility. Rigorous, independent, multi-center external validation is the non-negotiable minimum standard (120). The significant variability in MRI and PET parameters across institutions is a particularly acute bottleneck.

### 5.2 Beyond explainability: the need for causal AI and interpretable-by-design models

Clinicians are rightfully hesitant to trust “black box” algorithms (121, 122). While Explainable AI (XAI) techniques like Grad-CAM (123) and SHAP (124) provide *post-hoc* correlations, their value in high-stakes clinical decisions is fundamentally limited because they reveal correlation, not causation. These methods show *what* a model is looking at, but cannot guarantee *why* it is looking there (125). Critically, if a model has learned via a “shortcut,” XAI heatmaps may misleadingly highlight a confounding artifact, providing a false sense of security and an incorrect explanation to the clinician (126, 127).

The next frontier is therefore Causal AI, which represents a paradigm shift from asking “what” to asking “why” (128). The goal of Causal AI is to learn a model that reflects the true biological cause-and-effect chain. For instance, a causal model for HRD prediction would not merely correlate image texture with the label, but would learn to identify the specific imaging manifestations of disorganized neovasculature and necrosis that are *caused by* HRD-driven genomic instability. Such a model, grounded in causality, is inherently more robust to confounders and more trustworthy. Furthermore, this aligns with a growing movement towards interpretable-by-design (“white box”) models, which, as argued by proponents like Cynthia Rudin, offer a more reliable path to clinical trust than attempting to explain a “black box” *post-hoc* (126, 129).

### 5.3 The data bottleneck and the need for a gold standard validation

The biggest bottleneck remains the availability of high-quality, curated, multi-modal data governed by FAIR principles (130, 131). Moreover, for the digital biopsy paradigm to be validated, a new “gold standard” is required (132). Spatial Transcriptomics (ST) is the definitive technology for this (133, 134). By co-registering pre-operative imaging with post-operative ST data from the same tumor, one can directly verify whether a radiomic feature for “immune hot” truly corresponds to a region with high T-cell gene expression (134–136). ST is not just a research tool; it is the necessary ground truth for validating the biological basis of any digital biopsy model before clinical consideration (137).

This vision is no longer hypothetical but is being actively implemented in pioneering clinical trials. A perfect exemplar is the NCT06324175 (CO-MOULD) trial for high-grade serous ovarian cancer. This study directly tackles the core challenge of radiogenomic validation: achieving perfect spatial co-registration between *in vivo* imaging and *ex vivo* tissue analysis. The trial employs an innovative methodology: based on a patient’s pre-operative CT/MRI scans, a patient-specific 3D-printed mould of the tumor is created. After surgical resection, this mould acts as a precise cutting guide, allowing pathologists to slice the tumor along anatomical planes that perfectly correspond to the original imaging slices (e.g., axial plane) (138).

The significance of this technique is profound. It enables, for the first time, a direct, spatially-matched validation of whether a radiomic feature observed in a specific tumor “habitat” on an MRI scan truly corresponds to a specific gene expression profile (via ST) or histological pattern at that exact physical location. This represents a revolutionary leap from macro-level, whole-tumor correlation studies to micro-level, spatially-resolved ground-truth validation, setting a new, rigorous standard for the entire digital biopsy field (138).

### 5.4 Federated learning: collaborative innovation while protecting privacy

For rare diseases, federated learning provides an elegant solution (139). Enables multiple centers to collaboratively train a global model while never accessing private patient data (140, 141). Key challenge is to address data heterogeneity (non-IID data) across centers, a problem being addressed by emerging techniques like federated personalization (142–144).

### 5.5 Clinical integration and regulatory approval

An AI tool must integrate seamlessly into clinical workflows (145). As “Software as a Medical Device” (SaMD), diagnostic AI tools require rigorous regulatory approval from bodies like the FDA (146) (**Table 2**).

**Table 2:** Emerging AI paradigms and their link to specific biological problems.

AI paradigm	Core principle & rationale	Potential application linked to molecular events (from Table 1)	Advantages over current models	Key challenges
Foundation Models	Self-supervised pre-training on large unlabeled data, followed by few-shot fine-tuning (53, 54)..	Create a single, powerful model for gynecologic imaging that can be rapidly adapted to predict multiple rare molecular events.	Dramatically improves data efficiency and generalizability, overcoming the primary bottleneck of data scarcity.	High initial pre-training costs; ensuring fairness and avoiding bias from massive, uncurated datasets.
Vision Transformers (ViTs)	Takes image and models it as sequence of patches, using self-attention to model long-range, global dependencies (164, 165)..	Analyze tumor invasive front: Predict local invasion in cervical cancer by modeling long-range cell-stroma interactions (46).	Superior in capturing global context compared to CNNs’ limited receptive fields.	High computational cost; adapting to smaller medical datasets is a research focus (164, 166).
Graph Neural Networks (GNNs)	Models data as a graph (nodes & edges) to capture entity features and their relationships (49).	Model the TME: Predict ICI response by modeling spatial relationships between cancer and immune cells (links to dMMR/MSI & HPV) (51).	Explicitly models relationships and spatial heterogeneity, ideal for TME analysis (167).	Defining nodes/edges to accurately represent the TME is a non-trivial biological and computational challenge (167).
Causal AI	Aims to learn causal relationships (why) rather than just correlations (what) (125).	Develop robust biomarkers: Build models that predict HRD based on causal biological drivers, making them insensitive to scanner type (168).	Moves beyond correlation to causation, leading to more robust, generalizable, and trustworthy models.	Causal inference from observational data is extremely challenging; requires strong domain assumptions and new methodologies (169).
Digital Twins	Patient-specific computational models integrating all data to simulate disease and treatment response (155).	Personalized therapy simulation: In silico testing of PARPi vs. chemo for a patient with a specific HRD score and tumor phenotype (157, 170).	The ultimate paradigm for personalized medicine: optimizing therapy in a risk-free virtual environment (171).	Immense data integration and computational challenges; requires extensive biological validation; currently a long-term vision (172, 173).

## 6 Conclusion and future perspectives

Artificial intelligence is no longer a distant concept but an increasingly imminent clinical reality in gynecologic oncology (147–149). We are on the cusp of a paradigm shift: medical imaging is evolving from a qualitative, anatomical tool into a powerful, quantitative probe capable of non-invasively decoding the core biology of a patient’s cancer (150). We have moved beyond simple prognosis to inferring specific, actionable molecular targets directly from pixels.

The road ahead is paved with immense opportunity. The next wave of innovation will stem from foundation models (53, 151). These models are usually pre-trained on large, unlabeled data sets using self-supervised learning algorithms., such as contrastive learning (e.g., ConVIRT, MedCLIP), which learns rich visual representations by aligning paired images and their corresponding text reports (152). However, their development in medicine faces unique challenges, including: 1) the scarcity of large-scale, diverse, and publicly available clinical imaging datasets; 2) the immense computational resources required to train on 3D volumetric data like CT and MRI; and 3) significant regulatory and ethical hurdles related to fairness, bias, and patient privacy (153). The deep fusion of imaging with spatial omics and liquid biopsies, and widespread implementation of privacy-preserving federated learning will also drive innovation (154).

The ultimate vision is the creation of patient-specific “digital twins”—*in silico* models that integrate all longitudinal data to simulate disease progression and predict individual responses to therapies, enabling truly dynamic, personalized treatment plan selection in a risk-free environment (155–157). However, the path to this vision is fraught with immense challenges. These include the profound complexity of integrating multi-scale longitudinal data (from genomics to imaging), the difficulty of biologically validating that the virtual model accurately reflects *in vivo* processes, and formidable computational, ethical, and regulatory barriers (158). Despite these hurdles, preliminary successes are emerging. For instance, technologies like FarrSight^®^-Twin have demonstrated the ability to accurately replicate the results of real-world clinical trials *in silico* for cancers including ovarian cancer, suggesting that, while challenging, the digital twin is steadily moving from a purely conceptual future to a scientific reality.

Turning this vision into reality requires unprecedented collaboration. We must insist on the highest standards of scientific rigor, champion high-quality data sharing, and demand algorithmic transparency and causality (159, 160). Only then can we fully harness the power of AI to deconstruct the complexity of gynecologic cancers, pixel by pixel, and deliver on the ultimate promise of precision medicine for every patient (161–163).

## 7 Clinical implications of the digital biopsy paradigm

Clinical integration is envisioned as a multi-step workflow. Representative models like DeepHRD (175) have demonstrated the feasibility of predicting molecular status from histology, a concept now expanding to radiology. A potential workflow includes: 1) Diagnostic Triage (e.g., distinguishing benign/malignant ovarian cysts via CT (174)); 2) Risk Stratification (e.g., predicting cervical cancer prognosis via MRI (81)); and 3) Therapeutic Prediction (e.g., using “digital twins” to simulate PARP inhibitor response (170)), thereby guiding precision management.

Guiding precision therapy: The paradigm provides imaging-based evidence to select targeted therapies, such as identifying HRD status to inform the use of PARP inhibitors in ovarian cancer or predicting MSI-H status for immune checkpoint inhibitors in endometrial cancer.
Optimizing risk stratification: It offers a more nuanced view of disease progression beyond simple HPV DNA detection in cervical cancer by non-invasively predicting molecular events like viral integration.
Characterizing the tumor microenvironment: AI can delineate “hot” vs. “cold” immune landscapes to predict immunotherapy response, providing insights that transcend the tumor cell itself.
Personalizing radiotherapy: This technology enables “dose-painting” by mapping tumor hypoxia and helps mitigate harm by predicting patients at high risk for severe radiotherapy-related toxicity.
Enabling early response assessment: Through “delta-radiomics,” clinicians can assess therapeutic response much earlier than traditional criteria, allowing for timely adjustments to treatment plans.

## 8 Key takeaways for future research and clinical translation

Expanding AI from molecular profiling to diagnostic triage: Beyond predicting molecular status, AI should be leveraged for accurate differentiation between benign and malignant gynecologic lesions. Emerging studies already demonstrate exceptional performance (e.g., AUC 0.97 for tumoral vs. non-tumoral ovarian lesions on contrast-enhanced CT, with further subtyping of endometriosis-associated ovarian cancer at AUC 0.85) (174). Integrating this diagnostic capacity into clinical workflows will enable fertility-sparing decisions, reduce overtreatment, and optimize follow-up strategies, thereby extending the value of AI-driven imaging across the entire patient journey.
Prioritizing rigorous validation: The foremost challenge is model generalizability. Future research must prioritize rigorous, independent, multi-center external validation to combat “shortcut learning” and ensure that AI models are robust and reliable across different patient populations and imaging equipment.
Moving from correlation to causation: For AI to be trusted in high-stakes clinical decisions, the field must evolve from explainable AI (XAI), which only reveals correlations, to Causal AI. Developing models that learn the underlying biological cause-and-effect chains is essential for creating trustworthy and robust clinical tools.
Establishing a new gold standard: The clinical translation of digital biopsies requires an accepted “ground truth” for validation. Spatial transcriptomics, especially when combined with innovative co-registration techniques like the 3D-printed moulds used in the CO-MOULD trial, represents the necessary standard to biologically validate imaging-based predictions.
Harnessing next-generation AI: Foundation models, pre-trained on vast datasets, hold immense promise for overcoming data scarcity in medicine. However, their development requires addressing significant challenges related to data availability, computational cost, and ethical considerations such as fairness and bias.
Embracing collaborative and privacy-preserving models: Federated learning offers a critical solution to the data bottleneck. Fostering such collaborations is key to developing large-scale, diverse training datasets by allowing multiple institutions to train powerful models without compromising patient privacy.
Pursuing the “digital twin” as the ultimate goal: While a long-term vision, the patient-specific “digital twin” represents the pinnacle of personalized medicine, enabling *in silico* clinical trials to optimize therapy. Realizing this vision will require unprecedented interdisciplinary collaboration to overcome immense data integration, biological validation, and computational challenges.
